# Diagnosis and treatment of systemic vasculitis: Case report and literature review

**DOI:** 10.1097/MD.0000000000045708

**Published:** 2025-11-21

**Authors:** Aiying Liu, Lin Wang, Hong Li, Yue Meng

**Affiliations:** a Baotou Central Hospital, Baotou, Inner Mongolia, China.

**Keywords:** clinical picture, diagnose, medical record, systemic vasculitis, treat

## Abstract

**Rationale:**

Systemic vasculitis is one of the most challenging, complex and difficult diseases in the field of rheumatic diseases and even in the field of internal medicine, which can involve multiple systems of the whole body. Owing to their rich vascular beds, the lungs and kidneys are among the most commonly affected organs in systemic vasculitis. Lung involvement can manifest as interstitial lung disease, diffuse alveolar hemorrhage, pulmonary nodules, pulmonary arterial hypertension, pulmonary aneurysms, pulmonary arteriovenous thrombosis, etc, which are easily confused with infections, tumors, and other diseases. Renal involvement in systemic vasculitis presents with a diverse spectrum of clinical manifestations. Given its high prevalence, complex clinical presentation, significant diagnostic challenges, and generally poor prognosis, achieving a thorough understanding of its various phenotypes, establishing an early diagnosis, and initiating prompt and aggressive treatment are crucial for substantially improving patient outcomes.

**Patient concerns:**

A 68-year-old man presented with a cough. His condition deteriorated despite antibiotic treatment for suspected pneumonia, followed by the development of worsening renal function evidenced by progressively elevated serum creatinine and proteinuria. Subsequent serological testing was positive for antineutrophil cytoplasmic antibody antibodies.

**Diagnoses:**

Laboratory tests and pulmonary computed tomography scan.

**Interventions:**

The patient was started on a regimen of oral cyclophosphamide and high-dose glucocorticoids.

**Outcomes:**

The patient’s renal function has deteriorated and requires maintenance hemodialysis.

**Lessons:**

This case emphasizes the necessity of maintaining vigilance when distinguishing ANCA-associated vasculitis lung damage from lung infection in patients.

## 1. Introduction

Systemic vasculitis is a disease with a multifactorial and often incompletely understood etiology. Its heterogeneous clinical presentation poses significant challenges in both diagnosis and management, frequently leading to delayed recognition and an unfavorable prognosis. Therefore, a thorough understanding of its pathogenesis, diverse clinical features, and contemporary treatment strategies is essential for optimizing patient care.

## 2. Case presentation

The patient is a 68-year-old male who was admitted to the respiratory department of our hospital on March 6, 2024, due to “persistent cough for 20 days.” The patient developed coughing without obvious inducement, mainly at night, with a small amount of sputum and difficulty in expectoration, accompanied by acid reflux and heartburn, and occasional chest tightness. There was no fever, chills, or coldness, no abdominal pain or diarrhea, and no frequent urination, urgent urination, or dysuria. He self-medicated with licorice tablets (dose and frequency unknown) and other symptomatic treatments outside the hospital, but the symptoms did not improve. No laboratory tests were conducted. The community hospital considered it to be pulmonary inflammation and administered intravenous anti-inflammatory drugs (name, dose, and frequency unknown) for 3 days, but the cough did not subside.

**Figure 1.**
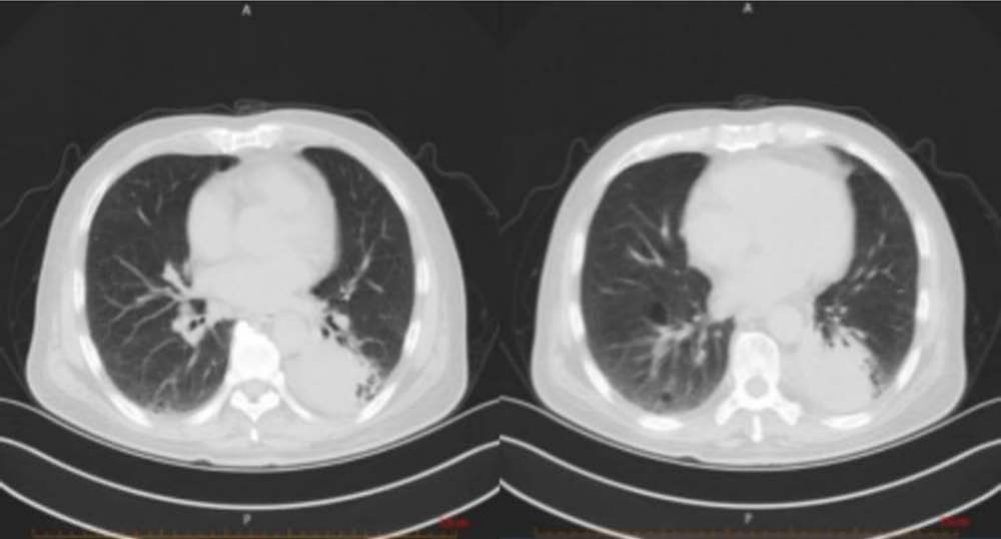
Lung CT on March 4th: the bronchi and vascular bundles of both lungs are thickened, with patchy translucent shadows and quasi-circular lung-free texture areas, patchy high-density shadows in the lower lobe of the left lung, partial localized stenosis in the lower lobe of the left lung, patchy ground glass-like density shadows and fibrous cord shadows in both lungs. CT = computed tomography.

On March 4th, he went to the respiratory clinic for a lung computed tomography (CT) scan, which showed bilateral interstitial changes and left lower lobe space occupying lesion with left pleural effusion (Fig. 1). The doctor believed it was a lung infection, and the patient was admitted to the respiratory department. After completing relevant laboratory tests and examinations in the respiratory department, pulmonary infection was considered. Administer 2.5 g of meropenem intravenously every 12 hours and 1.5 g of cefoperazone sulbactam intravenously every 8 hours to the patient for anti-inflammatory treatment.

Past history: in 2014, he underwent surgery for colon cancer; in 2017, due to lower limb skin purpura, the patient was diagnosed with allergic purpura at an external hospital and received oral hormone therapy (dosage and duration unknown), which improved the condition. At that time, the renal creatinine test was 167 μmol/L, and no systematic diagnosis or treatment was conducted; in 2018, the patient was admitted to the hospital with a creatinine level of 120 μmol/L, a 24-hour urine protein quantification of 0.46 g/h antineutrophil cytoplasmic antibody (ANCA) negative, and a creatinine clearance rate of 60.74 mL/min. The diagnosis was chronic nephritis syndrome. It was suggested to perform a kidney biopsy, but due to the invasive nature of the surgery, the patient refused and was given 1.68 g Jinshuibao capsules 3 times a day and 5 g uremic clearance granules 4 times a day for symptomatic kidney protection and detoxification treatment. There were no complaints of medication use, and the patient was not followed up afterwards. He denied having a history of hypertension, diabetes, and coronary heart disease.

After hospitalization, the patient’s renal function was monitored, and the serum creatinine was 575 μmol/L. After contacting our department for consultation for many times, combined with the patient’s medical history and past history, the patient was considered to have acute exacerbation of chronic renal insufficiency. During hospitalization, the patient was given active anti-inflammatory, fluid replacement, and other symptomatic treatments, however the serum creatinine of the patient still increased progressively, up to 857.2 μmol/L, and the urine output decreased significantly, 24 hours urine output was 500 mL.

After that, due to significant abnormal creatinine levels and reduced urine output in renal function, there are symptoms of heavy water load such as chest tightness and shortness of breath, our department gave central venous catheterization and began renal replacement therapy. During this period, the patient’s left lower lobe space-occupying lesions were considered as inflammatory lesions by perfect positron emission tomography-CT in outpatient department. After anti-inflammatory treatment with mezlocillin (2.5g q8h for 4 days and 2.5g q12h for 2 days), chest CT reexamination.

**Figure 2.**
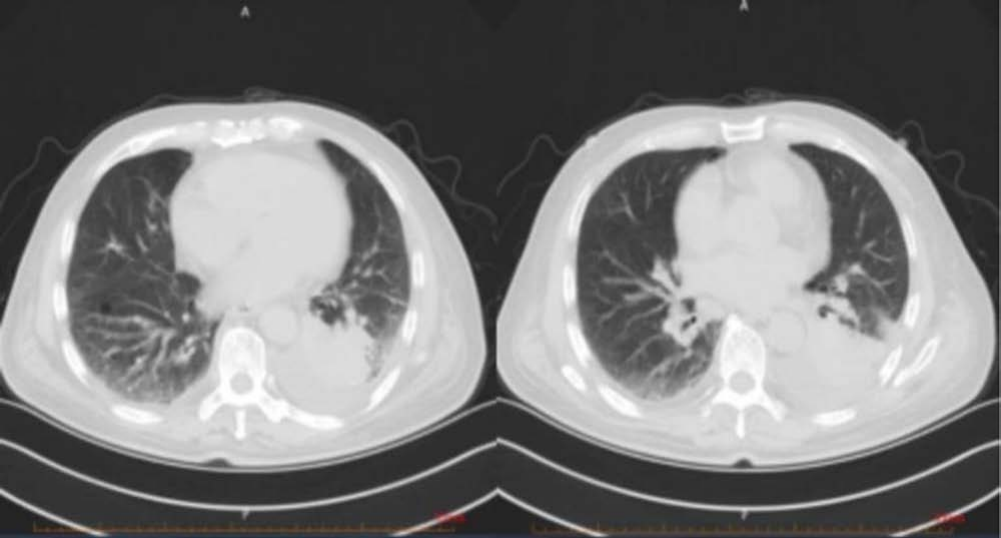
Lung CT on March 11th: compared with March 4th, the lung field transparency has increased significantly, and the exudative lesions in both lungs have become more severe. The range of patchy high-density shadows in the lower lobe of the left lung has slightly decreased compared to before. CT = computed tomography.

On March 11th showed that multiple fibrotic strips and exudative lesions in both lungs were aggravated, and the lesions in the left lower lobe were slightly reduced (Fig. 2).

Because the patient required maintenance hemodialysis treatment, she was recommended to continue anti-inflammatory treatment and transferred to our department on March 14th for further treatment of renal disease.

Physical examination after transfer: T 36°C, R 20 times/min BP 144/80 mm Hg, peripheral oxygen saturation (SpO2) is 96%, conscious, with symptoms of anemia, no eyelid edema, mild cyanosis of the lips, and no blowing of wind in the bilateral blood vessels of the neck. Bilateral auscultation shows rough breathing sounds without dry or wet rales. Physical examination revealed no evidence of bilateral lower extremity edema.

Auxiliary examination: blood gas analysis showed that: hydrogen ion concentration 7.368, partial pressure of carbon dioxide 29 mm Hg, partial pressure of oxygen 73 mm Hg, SpO2 93.8%, base excess −7.4 mmol/L, potassium 4.65 mmol/L, lactic acid 1.24 mmol/L, urine routine showed protein 3+, occult blood 3+; blood routine showed that the white blood cells were 7.74 × 10^9^/L, the red blood cells were 3.73 × 10^12^/L, neutrophil ratio 79.2%, and lymphocyte ratio 7.9%. C-reactive protein 209.16 mg/L, procalcitonin 0.476 ng/mL, B-type brain natriuretic peptide 206 pg/mL; urea 24.2mmol/L, creatinine 575 μmol/L; and antinuclear antibodies: anti-CENP B positive. Antinuclear antibody positive (1:100); fungal G tests were normal. The ANCA and anti-glomerular basement membrane antibody tests were negative after admission.

Initial diagnosis: chronic kidney disease stage 5, acute exacerbation of chronic renal insufficiency, hemodialysis, renal anemia, pneumonia, pleural effusion, emphysema, pulmonary bulla, hypoproteinemia, was given electrocardiogram monitoring, nasal catheter and mask oxygen inhalation, anti-inflammatory and renal replacement therapy.

**Figure 3.**
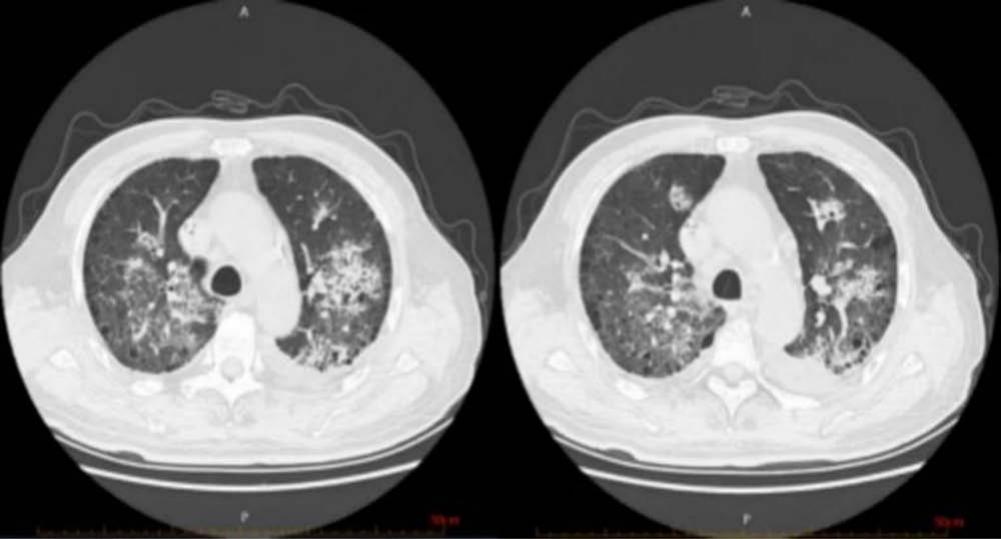
Lung CT on March 26th: compared with March 11th, multiple fibrotic streaks and exudative lesions in both lungs have significantly worsened compared before, while the patchy high-density shadow in the lower lobe of the left lung has slightly decreased compared before. CT = computed tomography.

Four days after the transfer, the patient developed blood phlegm, chest tightness, shortness of breath, and was unable to lie flat. The 24-hour urine output is about 400 mL, and the blood routine test shows that the red blood cell count is 2.81 × 10^12^/L and the platelet count is 97 × 10^9^/L, with N-terminal pro-brain natriuretic peptide‌>35,000 pg/mL. Our hospital’s antinuclear antibody (1:100) and anti-myeloperoxidase antibody (Immunoglobulin G type) are both positive, while enzyme linked immunosorbent assay and Western blot tests for ANCA are negative. The patient’s urine microscopic examination shows normal red blood cells, and there are no systemic lupus erythematosus specific antibodies or clinical manifestations. Additionally, the patient does not agree to undergo renal biopsy. Therefore, lupus nephritis and immunoglobulin A nephropathy induced acute progressive glomerulonephritis are difficult to consider. Due to poor anti-inflammatory treatment, the patient still experiences hemoptysis and rapid progression of renal function. We believe that ANCA related vasculitis and pulmonary edema cannot be ruled out, and therefore, enhanced blood purification and ultrafiltration dehydration treatment should be given. The patient’s symptoms of hemoptysis and shortness of breath have improved, and the oxygen saturation under anaerobic conditions is 87%. However, the patient still has hemoptysis, and some bowel sounds can be heard in the posterior part of both lungs. Pulmonary edema cannot fully explain the patient’s condition, and after consultation with the director of the respiratory department, opportunistic infections cannot be ruled out. Next-generation sequencing technology was suggested to further identify the pathogen, but patients and their families strongly opposed it. High resolution CT reexamination of the chest showed significant deterioration of multiple fibrous cords, exudative lesions, and patchy ground glass opacity in both lungs, with a slight decrease in patchy high-density shadows in the posterior basal segment of the left lower lobe compared to before (Fig. 3).

On March 27th, the difficult case was discussed and consulted by the director of imaging department. After reading the CT images, it was considered that it was consistent with vasculitis. Therefore, our department corrected the diagnosis of ANCA negative vasculitis, pulmonary vasculitis, pulmonary hemoptysis, renal vasculitis and renal failure. Immunomodulatory therapy with methylprednisolone 40 mg was given and anti-inflammatory therapy with prodoxycycline 0.1 g every 12 hours was continued. The patient’s hemoptysis symptoms did not alleviate, and gradually appeared blood system damage, manifested as anemia and significant thrombocytopenia. However, at this time, the reexamination of the patient’s D-dimer was 2085 ng/mL, and thrombosis could not be excluded, but no obvious thrombosis was found in the veins of the lower limbs.

Due to the obvious involvement of the lung, kidney and blood system, the patient’s condition was complex and critical, and intensified immunotherapy was started on March 28th. Considering the patient’s older age and suspected pulmonary infection, 80 mg methylprednisolone was started, at the same time, antiviral treatment with ganciclovir, doxycycline and compound sulfamethoxazole were given according to the consultation advice of relevant departments. However, the patient’s compliance was poor during the treatment, and doxycycline and compound sulfamethoxazole were not prescribed. After 2 days of treatment, the patient’s symptoms were not significantly relieved, and on March 30th, the patient developed hemoptysis (about 50 mL/d), chest obstruction, and shortness of breath again, which were more severe than before. Wet rales and velcro rales could be heard in both lungs. Blood gas analysis showed that the partial pressure of oxygen was 46.4 mm Hg, SpO2 was 81.2%, The patient coughed up blood in the early stage, considering that it was caused by water load center of gravity failure. However, after active ultrafiltration dehydration, the patient still had coughing up blood. Combined with CT reexamination, it was found that the lung lesions had worsened, with diffuse alveolar bleeding and hypoxemia. There were plasma exchange indicators, with a single plasma exchange of 2500 mL and methylprednisolone 400 mg pulse therapy. On the 2nd day, 320 mg methylprednisolone pulse therapy was given, and bedside renal replacement therapy was performed to actively ultrafiltration dehydration. The patient’s symptoms of cough, expectoration, and hemoptysis were better than before, and SpO2 in the high pillow position was maintained above 90%. On the 3rd day, methylprednisolone was adjusted to 240 mg.

**Figure 4.**
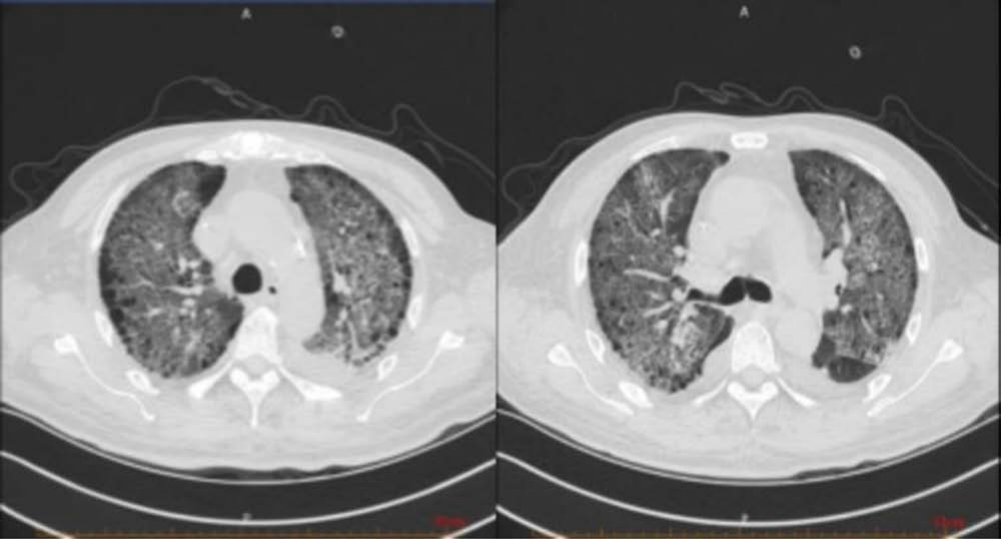
CT of the lung on April 2nd: compared with March 26th, the area of multiple exudative lesions in both lungs has increased compared to before, while the size of some lesions has decreased and the density has decreased. CT = computed tomography.

Following the aforementioned treatment regimen, the patient’s general condition improved, with a marked alleviation of symptoms including hemoptysis, cough, and sputum production. Reexamination of chest CT showed multiple exudative lesions in both lungs, which were larger than before, and some lesions were reduced in scope and density (Fig. 4). However, given the critical and complex nature of the patient’s condition, along with persistent uncertainty regarding further management, remote consultation was conducted with experts from the Department of Respiratory and Rheumatology at Beijing Chaoyang Hospital. Their assessment indicated insufficient evidence to support an immunological disease, and they recommended a lung biopsy to evaluate for possible pulmonary occupying lesions. The patient’s family was re-consulted regarding the procedure; however, they firmly declined lung biopsy. Given the significant clinical improvement following the previous treatment, and based on the overall diagnostic assessment, our department confidently concluded the diagnosis of ANCA-negative vasculitis. Treatment was therefore continued with oral methylprednisolone 40 mg once daily. Currently, the available symptomatic and imaging evidence was insufficient to support a diagnosis of pulmonary tuberculosis. However, given the intention to initiate prolonged high-dose glucocorticoid therapy, the Department of Infectious Diseases recommended prophylactic antituberculosis treatment. The patient demonstrated poor compliance and self-discontinued the antituberculosis medications after only 1 week.

**Figure 5.**
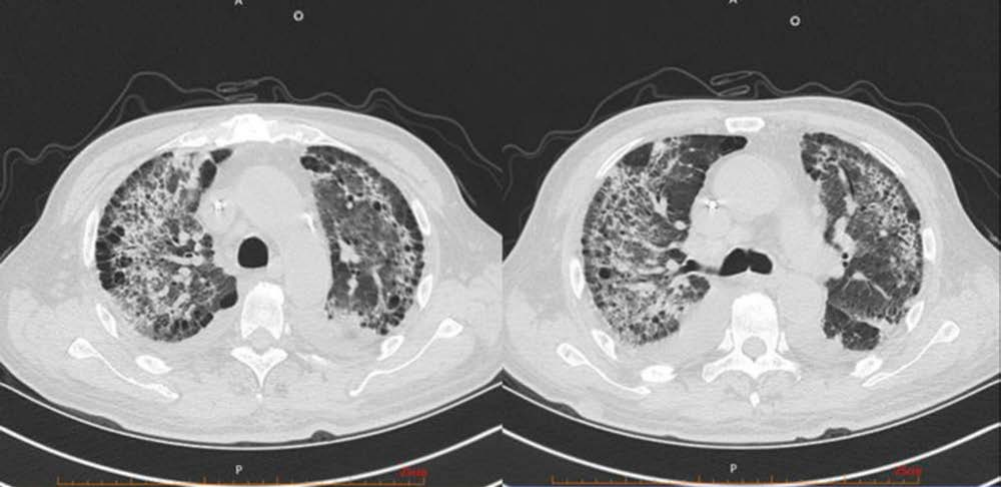
HRCT of the lung on April 11th: compared with April 2nd, multiple exudative lesions in both lungs are similar to those observed previously. HRCT = high-resolution computed tomography.

A reexamination of chest high-resolution computed tomography on April 11th showed that multiple exudative lesions in both lungs were similar (Fig. 5). SpO2 in sitting position could be maintained at 97% under mask oxygen inhalation with 4 L/min oxygen flow rate.

**Figure 6.**
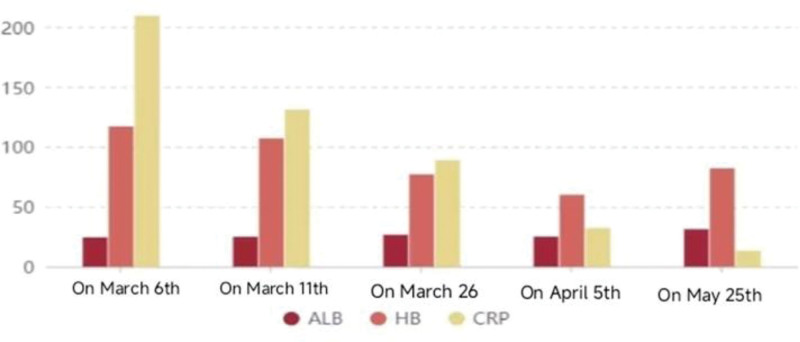
Bar chart of albumn (ALB), hemoglobin (HB), and C-reactive protein (CRP) changes in patients, CRP decreased significantly with treatment.

**Figure 7.**
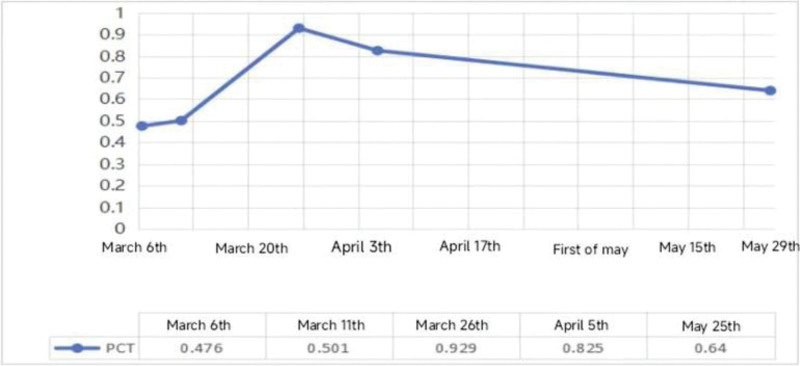
Trend chart of procalcitonin (PCT) changes in patients, it began to rise on March 6th, and reached the highest value of 0.929 on March 26th, and then gradually decreased.

Cyclophosphamide (CTX) 0.2 g ivgtt was added on April 23rd, and the patient’s condition was improved and discharged, but the intravenous infusion of CTX was inconvenient and the compliance was poor, so the intravenous infusion was changed to 50 mg every 3 days, and the methylprednisolone was adjusted to 32 mg/d. During the period, the C-reactive protein and procalcitonin showed a downward trend compared with before (Figs. 6–7).

On April 27th, the patient could walk without oxygen for more than 10 minutes, and SpO2 could be maintained 100% in the sitting position under mask oxygen inhalation with 5 L/min oxygen flow rate. At present, the patient’s cardiopulmonary function has improved significantly, and he can walk more than 1 hour on level ground, and his SpO2 can be maintained at 93% without oxygen inhalation, which further supports the diagnosis of ANCA-negative vasculitis. At present, the patient’s methylprednisolone has been reduced to 12 mg/d.

**Figure 8.**
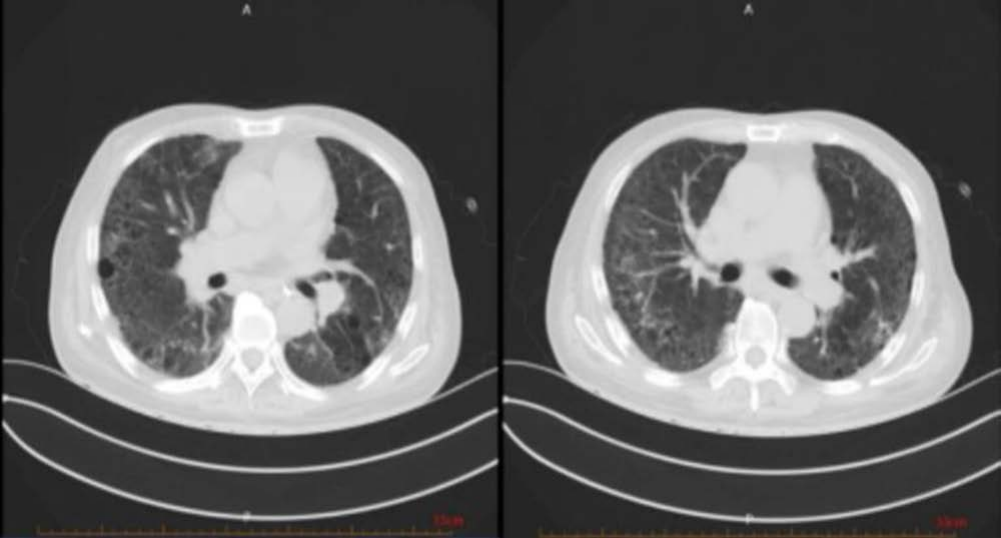
Lung CT on June 12th: compared with April 11th, multiple exudative changes in both lungs have decreased compared to before, and bilateral pleural effusion has basically disappeared compared to before.

**Figure 9.**
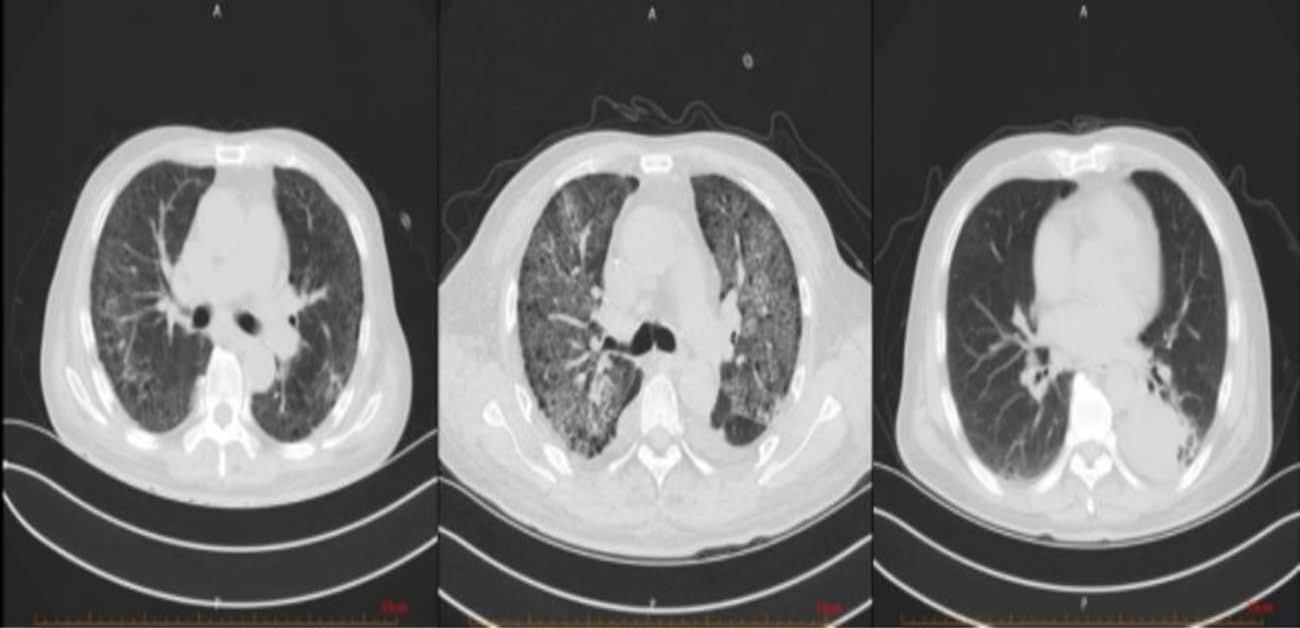
Lung CT contrast on March 4th, April 2nd, and June 12th: as the disease progressed and the diagnosis and treatment of lung lesions were carried out, the exudative lesions in the patient’s lungs decreased compared to before.

**Figure 10.**
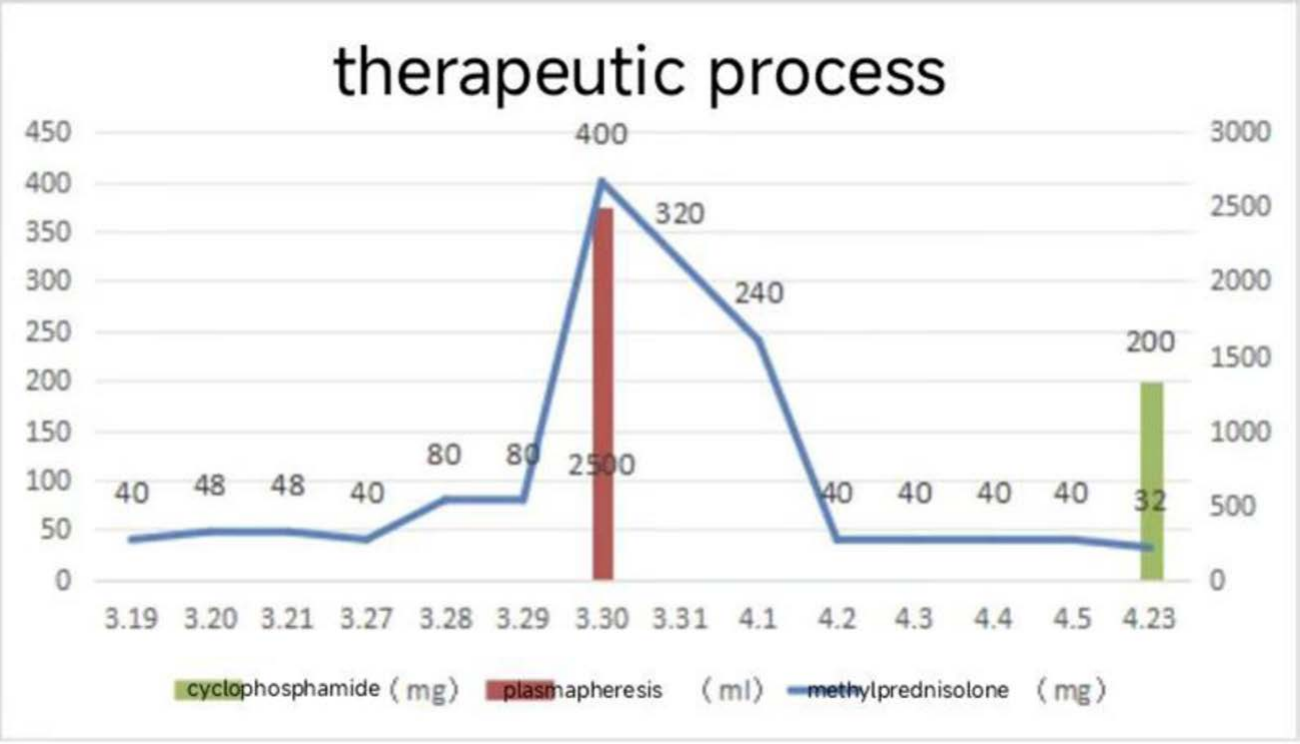
Therapeutic process, plasma exchange was performed on March 30th, cyclophosphamide was given on April 23rd, methylprednisolone treatment runs through.

Reexamination of lung CT on June 12th showed that multiple exudative lesions in both lungs were reduced compared with before (Figs. 8–9). The treatment was continued regularly with blood purification 3 times a week and oral treatment with methylprednisolone 16 mg once a day and CTX 50mg once every 3 days (Fig. 10).

## 3. Discussion

We report a case of ANCA-negative systemic vasculitis with both lung and kidney involvement. Systemic vasculitis is a large group of complex and heterogeneous diseases with vascular inflammation and necrosis as the main manifestations, which can involve multiple systems throughout the body^[1,2]^. First, Systemic manifestations: most patients have systemic symptoms such as fever, fatigue, loss of appetite and weight loss. Joint swelling and pain occur in 30% to 80% of ANCA-associated vasculitis (AAV) patients, and myalgia is also a common symptom in AAV patients. Second, Skin and mucosa: skin and mucosa are one of the most commonly affected organs of AAV. Thirty percent to 60% of granulomatosis with polyangiitis (GPA) patients, 40% to 70% of microscopic polyangiitis (MPA) patients and 51% to 67% of eosinophilic granulomatosis with polyangiitis (EGPA) patients have skin and mucosa lesions. The main clinical manifestations were oral ulcer, rash, purpura, livedo reticularis, skin infarction, ulcer and gangrene, and multiple fingertip ulcers were common. Third, Eye: 30% to 60% of GPA patients and 10% to 30% of MPA patients will have ocular involvement, but only <10% of EGPA patients will have ocular involvement. The common manifestations of ocular lesions included conjunctivitis, blepharitis, keratitis, scleritis, iritis, and prominent exophthalmos in some patients. Fundus examination can show retinal exudation, hemorrhage, vasculitis and thrombosis, and a few patients may have diplopia and decreased vision. Fourth, Ear, nose, and throat (ENT): ENT is a common site of AAV. Ent lesions occur in 80% to 90% of patients with GPA, 20% to 30% of patients with MPA, and nearly 53% to 70% of patients with EGPA. The most common ear involvement was otitis media, neurosensory, or conductive hearing loss. Auricle redness, swelling, heat, and pain may occur in ear cartilage involvement. Nasal obstruction, bloody purulent discharge, bloody nasal crust, hyposmia, or loss of smell are common manifestations of nasal and paranasal sinus inflammation. Nasal polyps are common nasal manifestations in patients with EGPA. Nasal cartilage involvement can lead to saddle nose; Involvement of laryngeal and tracheal cartilage may present with hoarseness, stridor, and dyspnea. Fifth, Respiratory system: respiratory system is one of the most commonly involved organs in AAV. The common manifestations of respiratory tract involvement were persistent cough, expectoration, wheezing, and hemoptysis and dyspnea in severe cases. Asthma is the earliest respiratory manifestation in patients with EGPA and can appear years before other manifestations. Pulmonary lesions can be manifested as infiltrates, multiple nodules, cavitation, and interstitial lesions on imaging. Sixth, Nervous system: nervous system is one of the most common organs involved in AAV, and peripheral nerve involvement is more common. Ten percent to 50% of GPA patients, 20% to 57% of MPA patients and 42% to 84% of EGPA patients will have peripheral neuropathy. Mononeuritis polyneuritis is the most common peripheral neuropathy. Patients may have numbness in the hands and feet, drop the wrist and foot, which seriously affects the patient’s mobility and quality of life, and is also one of the manifestations of the disease. A minority of patients can also develop peripheral sensory neuropathy, which manifests as paresthesia. Central nervous system involvement can be manifested as confusion, convulsions, stroke, and encephalomyelitis. Seventh, Kidney: kidney is one of the most common organs involved in AAV. The common manifestations of renal involvement were hematuria, proteinuria, edema, and hypertension, especially hematuria. Patients with severe renal function damage have increased serum creatinine, and some patients will have rapidly progressive renal failure. Eighth, Heart: although cardiac involvement in AAV is not common, it is closely related to the prognosis of patients. Approximately 5% to 15% of patients with GPA and 10% to 20% of patients with MPA will develop cardiac involvement, while 22% to 49% of patients with EGPA will develop cardiac lesions. Cardiac involvement can be manifested as pericarditis, pericardial effusion, cardiomyopathy, and heart valve insufficiency. Coronary artery involvement can occur in some patients, presenting with angina pectoris and myocardial infarction. Ninth, Abdomen: although abdominal involvement is rare in AAV patients, which only occurs in about 10% to 30% of patients, it is an important factor for poor prognosis. Abdominal involvement manifests as abdominal pain, diarrhea, bloody stools, bowel perforation, bowel obstruction, peritonitis, and, in a small number of patients, acute pancreatitis can also occur.

In this case, the patient presented with predominantly pulmonary and renal involvement. The lungs, which possess an extensive vascular network, are among the most frequently affected organs in systemic vasculitis. The clinical manifestations were dry cough, shortness of breath after exercise, and dyspnea. Chest CT showed ground glass opacity, interlobular septal thickening, vascular and bronchial wall thickening in the early stage, honeycombing lung and traction bronchiectasis in the late stage. Usual interstitial pneumonia and nonspecific interstitial pneumonia are the most common pathological types, and usual interstitial pneumonia is associated with poor prognosis.^[3]^ Pulmonary function tests showed restrictive ventilatory dysfunction and decreased CO diffusion capacity. The pathological finding was fibroblastic granuloma in the alveolar space Form. In recent years, it has been greatly developed to assess vascular involvement, disease activity and severity, and plays an important role in the diagnosis and treatment of vasculitis. X-ray, CT, ultrasound, Angiography, right heart floating catheter, CT Angiography, MR Angiography, MR Venography, positron emission tomography-CT.^[2]^

Pulmonary involvement in systemic vasculitis represents a serious and challenging clinical manifestation, necessitating a multidisciplinary approach to management. The appropriate treatment plan should be selected according to the severity of the disease, including induction of remission, maintenance, and symptomatic treatment. First, remission induction: glucocorticoids, CTX, biologics, immunoglobulin, and plasma exchange. Glucocorticoids have anti-inflammatory and immunosuppressive effects, and the appropriate dose should be selected according to the extent of lung involvement. CTX is the 1st choice of immunosuppressant to control disease progression. According to the 2021 American College of Rheumatology guidelines, rituximab is superior to CTX for remission induction in patients with severe GPA/MPA.^[4]^ Immunoglobulin is indicated for refractory pulmonary vasculitis or co-infection. Plasma exchange is often used as an adjunct to immunosuppressive therapy. Treatment recommendations in “Chinese Guidelines for the Diagnosis and Treatment of antineutrophil cytoplasmic antibody-associated glomerulonephritis (AAGN),”^[5]^ induction phase: for severe antibody-associated glomerulonephritis, treatment with glucocorticoids in combination with CTX, or with rituximab and CTX, is recommended. Second, maintenance therapy: low-dose glucocorticoid combined with immunosuppressants, including azathioprine, methotrexate, leflunomide, mycophenolate mofetil, etc. In cases of disease recurrence, prompt evaluation for underlying causes – such as co-infection or inadequate initial treatment – is essential, and management should be tailored to the severity of the relapse. Third, for the management of interstitial lung disease, antifibrotic agents such as pirfenidone and nintedanib may be considered.^[6]^ PAH can be treated with calcium channel blockers, endothelin receptor antagonists (bosentan, etc), prostacyclin analogues (iloprost, etc), phosphodiesterase type 5 inhibitors (sildenafil, etc), and guanylate cyclase agonists (riociguat, etc). The risk of pulmonary artery aneurysm or pulmonary artery thrombosis should be fully weighed, and surgery or anticoagulant therapy should be considered under active treatment such as immunosuppressive agents.^[7]^

The kidneys are highly vascular organs. Their blood supply originates from the abdominal aorta via the left and right renal arteries, which enter the kidneys and divide into several segmental arteries. These further branch into interlobar arteries, then arcuate arteries, and subsequently into interlobular arteries. From the interlobular arteries, afferent arterioles arise and supply the glomerular capillary tufts. The efferent arterioles then exit the glomeruli and give rise to the peritubular capillary network, which ultimately drains into the interlobular veins. The venous blood ultimately returns to the inferior vena cava through a series of draining veins. This elaborate vascular architecture – composed of successively branching and converging arteries and veins – is essential for supporting normal renal physiology. It also explains why the kidneys are among the most frequently affected organs in systemic vasculitis. Renal involvement represents the most prevalent and severe manifestation of systemic vasculitis, with an incidence exceeding 90%. It may occur in isolation without involvement of other organs. Common clinical features include proteinuria, hematuria, various types of casts, edema, and renal hypertension. Some patients develop impaired renal function, which may progress to end-stage renal disease. Systemic vasculitis can lead to multisystem damage and dysfunction. While pulmonary and renal involvement are most frequently observed, the disease may also affect other organs including the ENT, eyes, joints, cardiovascular system, nervous system, gastrointestinal tract, and skin. Furthermore, the clinical presentation of systemic vasculitis is often nonspecific, which contributes to a high risk of underdiagnosis or misdiagnosis.^[8–18]^

## Acknowledgments

We thank the patient for agreeing to clinical examination and the use of data.

## Author contributions

**Writing – original draft:** Lin Wang, Hong Li.

**Writing – review & editing:** Aiying Liu, Yue Meng.
